# Comparison of qPCR with ddPCR for the Quantification of JC Polyomavirus in CSF from Patients with Progressive Multifocal Leukoencephalopathy

**DOI:** 10.3390/v14061246

**Published:** 2022-06-08

**Authors:** Nyater Ngouth, Maria Chiara Monaco, Lorenzo Walker, Sydney Corey, Ijeoma Ikpeama, Gary Fahle, Irene Cortese, Sanchita Das, Steven Jacobson

**Affiliations:** 1 Viral Immunology Section, National Institute of Neurological Disorders and Stroke, National Institutes of Health (NIH), Bethesda, MD 20892, USA; nyater.ngouth@nih.gov (N.N.); monaco@ninds.nih.gov (M.C.M.); 2 Department of Laboratory Medicine, National Institutes of Health (NIH), Bethesda, MD 20892, USA; lorenzo.walker@nih.gov (L.W.); ijeoma.ikpeama@nih.gov (I.I.); gfahle@verizon.net (G.F.); sanchita.das@nih.gov (S.D.); 3 Neuroimmunology Clinic, National Institute of Neurological Disorders and Stroke, National Institutes of Health (NIH), Bethesda, MD 20892, USA; sydney.corey@nih.gov (S.C.); corteseir@ninds.nih.gov (I.C.)

**Keywords:** progressive multifocal leukoencephalopathy (PML), digital droplet PCR (ddPCR), cerebrospinal fluid (CSF)

## Abstract

Background: Lytic infection of oligodendrocytes by the human JC polyomavirus (JCPyV) results in the demyelinating disease called progressive multifocal leukoencephalopathy (PML). The detection of viral DNA in the cerebrospinal fluid (CSF) by PCR is an important diagnostic tool and, in conjunction with defined radiological and clinical features, can provide diagnosis of definite PML, avoiding the need for brain biopsy. The main aim of this study is to compare the droplet digital PCR (ddPCR) assay with the gold standard quantitative PCR (qPCR) for the quantification of JC viral loads in clinical samples. Methods: A total of 62 CSF samples from 31 patients with PML were analyzed to compare the qPCR gold standard technique with ddPCR to detect conserved viral DNA sequences in the JCPyV genome. As part of the validation process, ddPCR results were compared to qPCR data obtained in 42 different laboratories around the world. In addition, the characterization of a novel triplex ddPCR to detect viral DNA sequence from both prototype and archetype variants and a cellular housekeeping reference gene is described. Triplex ddPCR was used to analyze the serum from six PML patients and from three additional cohorts, including 20 healthy controls (HC), 20 patients with multiple sclerosis (MS) who had never been treated with natalizumab (no-NTZ-treated), and 14 patients with MS who were being treated with natalizumab (NTZ-treated); three from this last group seroconverted during the course of treatment with natalizumab. Results: JCPyV DNA was detected only by ddPCR for 5 of the 62 CSF samples (8%), while remaining undetected by qPCR. For nine CSF samples (15%), JCPyV DNA was at the lower limit of quantification for qPCR, set at <250 copies/mL, and therefore no relative quantitation could be determined. By contrast, exact copies of JCPyV for each of these samples were quantified by ddPCR. No differences were observed between qPCR and ddPCR when five standardized plasma samples were analyzed for JCPyV in 42 laboratories in the United States and Europe. JCPyV-DNA was undetected in all the sera from HC and MS cohorts tested by triplex ddPCR, while serum samples from six patients with PML tested positive for JCPyV. Conclusion: This study shows strong correlation between ddPCR and qPCR with increased sensitivity of the ddPCR assay. Further work will be needed to determine whether multiplex ddPCR can be useful to determine PML risk in natalizumab-treated MS patients.

## 1. Introduction

JCPyV, named with the initials of the patient with Hodgkin’s lymphoma from whom it was first isolated [1], is responsible for progressive multifocal leukoencephalopathy (PML), a rare and often fatal brain infection. PML develops when the virus infects oligodendrocytes, most frequently in patients with compromised immune systems [2,3] and, as more recent studies have reported, in patients treated with immunomodulatory drugs [4]. JCPyV can also infect neurons, leading to distinct clinical manifestations, such as granule cell neuronopathy of the cerebellum [5] and encephalopathy comprising cortical pyramidal neurons [6]. The diagnosis of PML, and of the less common manifestations of JCPyV infection, relies on demonstration of JCPyV either directly in brain tissue or in cerebrospinal fluid (CSF).

JCPyV has a circular, supercoiled, double-stranded DNA genome characterized by a hypervariable non-coding regulatory region (NCCR) located between the early and late protein-coding regions [7]. The precise nucleotide sequence of the NCCR is used to discriminate between different JCPyV variants. The variant excreted in the urine, known as the “archetype”, is considered the non-pathogenic JCPyV form because it has rarely been associated with PML, while the one labeled “prototype” is the pathogenic variant. The prototype and prototype-like forms derive as the result of serial deletions and duplications of unique nucleotide sequences in the NCCR of the archetype variant [8]. On the other hand, all JCPyV variants are characterized by the presence of a conserved T protein coding sequences. These specific features of the NCCR, coupled with the invariant T antigen region, allow the design of precise primers and probes that simultaneously amplify both the archetype and prototype variants of the JCPyV genome [9]. Therefore, together with MRI brain images and clinical assessments, the detection of JCPyV DNA in the CSF by PCR is an important diagnostic tool.

The distinct precision of quantitative PCR (qPCR) to target those unique sequences within both the NCCR and the JCPyV- large T antigen has made qPCR the “gold standard” for detection of the virus in human clinical samples. Although, the standard qPCR typically uses only large T antigens to measure JCPyV copy number as a laboratory diagnostic marker for PML. While qPCR is currently one of the best-known methods for quantification of viruses in biological samples [10]; this technique has inherent limitations that may preclude precise and accurate quantification of viral load [11]. Specifically, the dependence of quantification on extrapolation from a standard curve often results in high inter-assay variability and, at times, difficulty in accurately measuring viral load in compartments with low numbers of cells, such as the CSF [12]. Additionally, the standards are difficult to derive and maintain and do not often reflect the variability seen in a clinical specimen matrix [13,14].

Advances in PCR technology have introduced a third-generation PCR, the droplet digital PCR (ddPCR) that has the benefit of quantifying an absolute copy number independently of a standard [15]. A restriction enzyme is initially used to fragment the DNA in each sample, then those samples are mixed with the specific primers, fluorescent probes and other conventional PCR components. This mixture is combined with oil resulting in its partitioning into thousands of nanoliter-sized droplets. Each droplet is subsequently amplified by a thermocycler. All droplets are then queried for fluorescent signal ([15]). Each independent event (droplet) is defined as either positive or negative for the target probe by the amplitude of its recorded fluorescence signal [16]. Due to the random, independent segregation of DNA fragments into droplets, Poisson algorithms can be used to determine absolute copy numbers in the original sample independently of a standard curve [15]. The high sensitivity of ddPCR for the detection and quantification of clinically relevant pathogens could be of great value, especially when the quantity of nucleic acids is very low [16,17,18,19].

In this paper, we directly compared JC viral loads by standard qPCR, run in the NIH Clinical Center Microbiology Service, with ddPCR in CSF samples from PML patients, assayed simultaneously. Furthermore, we validated ddPCR precision and reproducibility by the direct comparison of ddPCR results with standardized JCPyV DNA analyzed by qPCR in 42 laboratories around the world. Lastly, we describe a novel triplex ddPCR to detect at the same time the non-pathogenic and pathogenic variants of JCPyV together with a cellular housekeeping reference gene used as an indicator of DNA quality and cellular quantity. 

## 2. Materials and Method

### 2.1. Clinical Samples

A total of 62 CSF samples from 31 patients with PML (see Table 1), collected between 2019 and 2021, were analyzed to compare the quantitative qPCR gold standard technique with ddPCR using primer sets and fluorescent probes to detect conserved viral DNA sequences in the JCPyV genome. A total of 60 serum samples were also included in this study. Serum samples were derived from different cohorts: 20 serum samples were collected from healthy controls, 20 serum samples were collected from treatment-naive patients with MS and 14 serum samples were collected from patients with MS undergoing immunomodulatory treatment with the anti-alpha 4-integrin monoclonal antibody, natalizumab (NTZ; the mean for NTZ-treatment duration was 22.6 months, with a range of 7–48 months). Three of the patients with MS being treated with NTZ seroconverted during the period of study from JCPyV-antibody negative to positive, with antibody titer ranging from low to high, as determined on routine clinical testing. An additional 6 serum samples obtained from PML patients not in the main cohort were also collected. All patients were enrolled in IRB-approved natural history studies and provided written informed consent for participation.

DNA was extracted from five plasma clinical samples and were sent blinded and coded to 42 different clinical laboratories in Europe and in the United States participating in a proficiency testing survey for quantitative PCR assay of JCPyV. The same DNA samples were used in ddPCR assay to amplify JCPyV (Table 2). A total of 12 CSF from non-PML patients were also used in this study (Supplementary Table S1).

A urine sample from a PML patient was used as positive control in all the ddPCR assays because of the genotypic characteristic of the JCPyV present in the urine, that has a unique 267 base pair arrangement in the NCCR together with the conserved T coding region.

### 2.2. DNA Extraction and Droplet Digital PCR

DNA was extracted from 200 µL of CSF and sera samples using the QIAamp MiniElute Virus Kit (Qiagen, Hilden, Germany) according to the manufacturer’s instructions. All DNA samples were eluted in 25 µL of elution buffer. Primers and probes sequences targeting the large T in JCPyV prototype and archetype variants and primers, and probes targeting the 66 bp sequence section of the NCCR characteristic of the archetype variant were used for ddPCR of JCPyV amplification (Supplementary Figure S1) [9]. The probe for the large T antigen was labeled with FAM and used at 20×, while those for the NCCR and the RPP30 housekeeping gene [16,20] were labeled with VIC but used at two different concentrations, 5× and 20× respectively. The primers and probe used for JCPyV-T-Ag have been published previously and were shown to be specific for JCPyV and do not cross react with BKV [21]. 10 µL of DNA was digested in a 1:1 ratio with digestion mix composed of HindIII restriction enzyme and 10× of its restriction enzyme buffer (New England BioLabs, Ipswich, MA, USA) for 30 min at 37 °C on a shaker. Because of its structural complexity, BioRad recommends the use of the restriction enzyme when using more than 66 ng of genomic DNA. This is further important for copy number analysis, ensuring that tandem repeats are separated [17]. After incubation, the digested DNA was diluted 1:2.5 with 30 µL of water and then, 20 µL of the digested and diluted DNA was used for the preparation of droplets. ddPCR was carried out using BioRad system and reagents as previously described [17]. Droplets were then moved to a 96-well PCR plate and amplified on a thermal cycler (GeneAmp PCR System 9700; Applied Biosystems, Waltham, MA, USA) with the following conditions: 95 °C × 10 min (1 cycle); 94 °C × 30 s, 59 °C × 1 min (40 cycles); 98 °C × 10 min (1 cycle) ending at 12 °C. After amplification, the plate was transferred into the QX200 droplets reader (BioRad, Hercules, CA, USA). Data were analyzed with QuantaSoft analysis and the quantification of the JCPyV target was calculated as copy number per milliliter of the initial specimen.

### 2.3. ddPCR Data Analysis

The multiplex (including triplex) ddPCR assay was optimized on the QX200 Droplet Digital system (Bio-Rad Laboratories). To evaluate the performance of the quantification of JCPyV copies, we used DNA extracted from the urine of a PML patient and used it as our positive control. The archetype variant excreted in the urine contains both the conserved viral DNA sequence in the large T-Ag coding region, and a 66 bp sequence in NCCR specific of the archetype variant. Thresholds were established manually for each experiment, based on negative controls, which included a no template control and a negative sample. Negative controls were included in each experiment. Droplet positivity was established by fluorescence intensity with a droplet count of >10,000 droplet sets as the cutoff for the analysis of all the ddPCR experiments. Data from any well with <10,000 droplets were discarded, and the experiment was repeated. The average total number of droplets generated was approximately 30,000. The experiment was also replicated if only two or fewer positive droplets were visualized. All samples were run in duplicate, and the final copy number is the average of the two measurements.

### 2.4. qPCR Analysis

The JCPyV real-time PCR assay performed at the NIH Clinical Center Microbiology Service targets a 78 base pair region of the JCPyV large T antigen gene [9] using the ABI 7500 Real-Time PCR System. This PCR system utilizes a single probe specific for the JCPyV sequence between the forward and reverse PCR primer sites [9]. A standard curve consisting of a dilution series of JCPyV cloned into a pCR2.1 plasmid is performed with each assay run. The cycle number (Ct) value of a positive sample is plotted on this curve to determine the number of target copies present in the reaction. Quantitative results for a positive specimen may only be reported if the value falls within the analytical measurement range (AMR) of the standard curve. If the positive value falls either above or below this range, the specimen is reported as positive but without a calculated numeric value. Reportable quantitative results are converted from the determined “copies/reaction” to “copies/mL”.

### 2.5. Statistics

Statistical analysis was preformed using GraphPad Prism. Paired *t*-test was applied for parametric variables and the Mann–Whitney test was also calculated when applicable.

## 3. Results

### 3.1. Characterization of ddPCR for the Detection of JCPyV-Tag and NCCR

**Figure 1:**
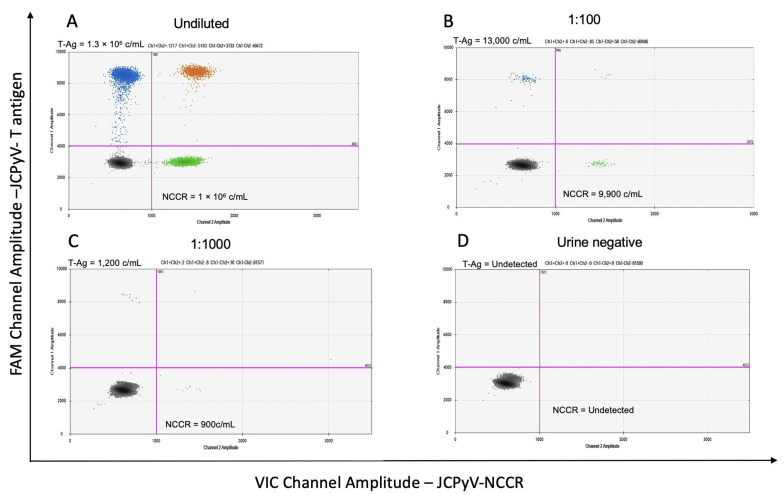
Representative two-dimensional ddPCR plots of a PML urine sample at different serial dilutions. (**A**) Urine DNA undiluted displays both JCPyV-large T-Ag and a sequence in the NCCR of the archetype at similar copies number value demonstrating the presence of only the non-virulent variant. (**B**,**C**) are serial dilutions of the same clinical samples. (**D**) No JCPyV genomes were detected in the urine sample of another PML patient.

This assay was designed to multiplex the conserved large T antigen sequence and the 66 bp sequence in the NCCR of JCPyV such that coinfection with prototype and archetype variants could be clearly quantified in clinical samples at the same time. A urine sample was used as positive control to detect and quantify viral DNA from both large T protein and NCCR sequences. Urine specimens are optimal to characterize the assay because of their “archetype” JCPyV genotype. Figure 1A–D are representative two-dimensional plots of DNA extracted from a PML patient’s urine sample. JCPyV-large T-Ag FAM is on the y-axis and JCPyV-NCCR VIC is on the x-axis. Population double positive for large T-Ag and NCCR is located in the upper right quadrant. In Figure 1A, the clinical sample was run undiluted. Both large T-Ag and NCCR show similar copy numbers (1.3 × 10^6^ and 1 × 10^6^ copies/mL, respectively), demonstrating that only the archetype, non-virulent variant, is present in this clinical sample. Figure 1B,C are serial dilutions of the same urine samples showing reduction in the positive populations without interference with the detection of the two JCPyV probes. As a negative control, Figure 1D shows an example in which no JCPyV genome was detected in the urine sample of another PML patient.

**Figure 2:**
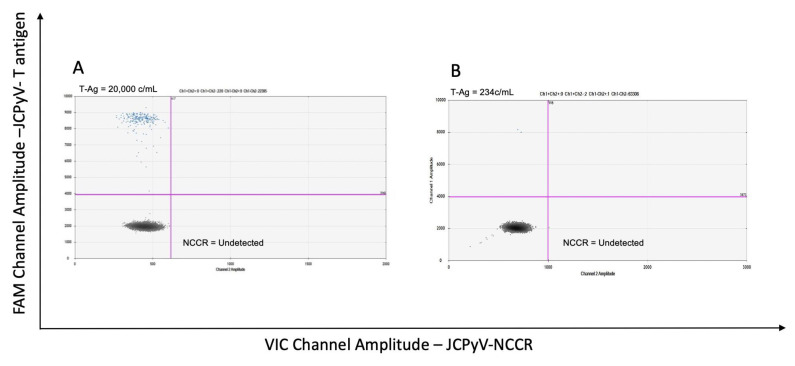
Representative two-dimensional plots of multiplex ddPCR. (**A**) CSF samples from a PML patient that demonstrated the presence of only JCPyV prototype at relatively high JCPyV copy number. (**B**) CSF from another PML patient as an example of a positive run at the lower limit of detection of the assay.

Figure 2A,B are representative plots of CSF samples from two patients with PML. Samples were considered positive if they showed two or more droplets at the expected amplitude set by the positive control. The sample shown in Figure 2A demonstrated only JCPyV prototype at relatively high copy number (20,000 copies/mL), while the PML CSF sample in Figure 2B had 234 copies/mL of the JCPyV prototype, within the lower limit of detection of the assay.

### 3.2. Comparison between qPCR and ddPCR

**Figure 3:**
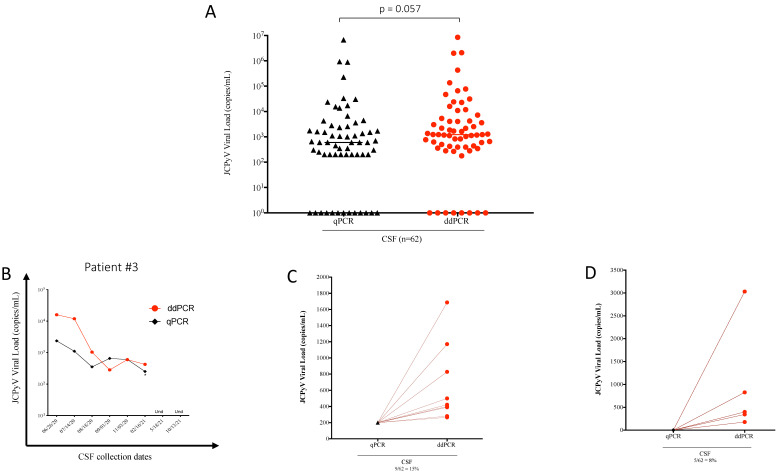
(**A**) Sixty-two JCPyV-DNA from CSF of PML patients at different time points amplified by qPCR and ddPCR show no significant difference between the two assays. (**B**) Representative plot comparing DNA samples extracted from CSF of a PML patient at 8 different collection dates and amplified by qPCR and ddPCR; * indicates limit of quantification for qPCR set at <250 copies/mL. The last two time points were undetected in both systems. (**C**) Representation of nine samples that were at the lower limit of quantification set at <250 c/mL by qPCR, while an absolute quantification could be determined by ddPCR. (**D**) Representation of five samples that were only detected by ddPCR. Limit of quantification for ddPCR = 110–250 copies/mL.

The comparison between qPCR and ddPCR was carried out in two ways: using patient CSF samples from which DNA was simultaneously amplified by the two technologies and using the same DNA from plasma samples comparing ddPCR quantitative values with qPCR cumulative results from 42 different laboratories worldwide. CSF samples from 31 PML patients at multiple time points for a total of 62 samples were used to compare qPCR and ddPCR technologies. qPCR and ddPCR showed comparable results and analysis of the values between the two assays did not reach statistical significance (*p* = 0.057; Figure 3A). Similarly, longitudinal samples from individual patients quantified using both assays yielded comparable results at each time point, with JC viral loads trending in same direction over time, as exemplified in Figure 3B. Supplementary Table S2 provides a comparison of the detectability of ddPCR and qPCR, and Supplementary Figure S2 is a scatter plot depicting the agreement between the two assays.

The lower limit of detection in qPCR was set at less than 250 copies/mL, considered to be positive but too low for accurate quantitation. The lower limit of detection in ddPCR was set at two droplets that correspond to approximately 110–250 copies/mL (Figure 2B). ddPCR could quantify JC viral loads from 9 of the 62 PML CSF samples (Figure 3C), which had qPCR values less than the lower limit of detection and therefore no relative quantitation could be determined by qPCR. By contrast, all these samples were positive by ddPCR with values that ranged from 200 to 2000 JCPyV copies/mL (Figure 3C). More importantly, of the original 62 PML CSF samples (Figure 3A; Supplementary Figure S2), 13 were negative by qPCR, but 5 of these 13 were determined to be JCPyV positive by ddPCR (Figure 3D; Supplementary Table S2; and Supplementary Figure S2, green dots). In the remaining 8 of the 13 CSF samples, JCPyV genome was undetected by both qPCR and ddPCR (Supplementary Table S2 and Figure S2, red dots). Furthermore, all 12 CSF samples from non-PML patients (Supplementary Table S1) were JCPyV undetected (data not shown). In addition, and as part of our validation process, five DNA clinical samples extracted from plasma were used as reference material sent blinded and coded to 42 different clinical laboratories in Europe and in the United States participating in a proficiency testing survey for quantitative PCR assay of JCPyV. As shown in Table 2, comparable results were obtained by qPCR and ddPCR (*p* = 0.46).

### 3.3. ddPCR Triplex Assay

**Figure 4:**
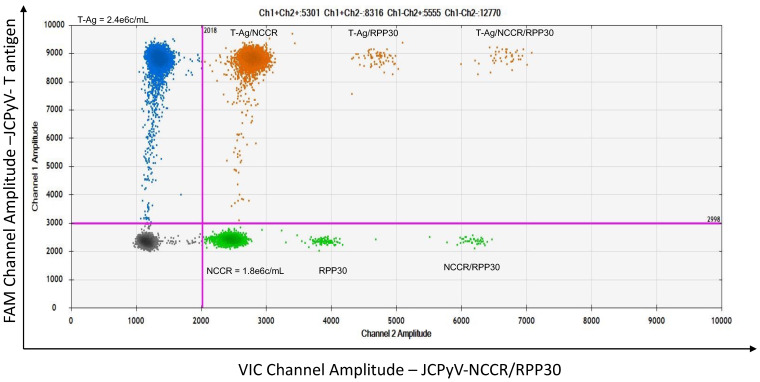
Representative two-dimensional plot of triplex ddPCR. Urine sample from a PML patient used as positive control. Primer sets and fluorescent probes to detect a conserved sequence in the JCPyV large T antigen FAM (y-axe; upper left quadrant; blue droplets) and a unique sequence of JCPyV-NCCR VIC for the archetype variant (x-axe; lower right quadrant; green droplets) and RPP30 VIC, housekeeping gene (x-axe; lower right quadrant; green droplets). By differing the primers/probe concentrations, RPP30 (20×) and NCCR (5×) could be used simultaneously on the VIC channel. Populations double positive for T-Ag, NCCR and RPP30 are in the upper right quadrant (orange droplets) and in the lower right quadrant (green droplets).

**Figure 5:**
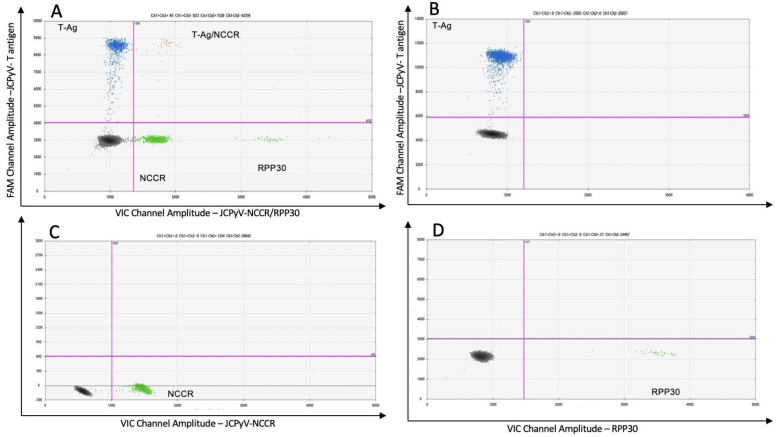
Representative plots of triplex and singleplex ddPCR of a CSF sample from a PML patient. (**A**) Triplex for JCPyV large T antigen, JCPyV-NCCR for the archetype variant and RPP30, housekeeping gene. Population double positive for T antigen and NCCR is in the upper right quadrant. (**B**) The FAM channel is on the y-axe, and the droplet population in the upper left quadrant reflects a positive detection of the Large T antigen coding region. (**C**) The VIC channel is on the x-axe detecting the unique sequence of the NCCR of the non-pathological JCPyV variant, and the droplet population in the lower right quadrant indicates a positive detection of this sequence. (**D**) The VIC channel targeting the house-keeping gene RPP30 is on the x-axe, and the positive droplet population in the lower right quadrant.

We used a multiplex format to detect, at the same time and in the same clinical samples, JCPyV large T-Ag and a unique sequence in the archetype NCCR, allowing the characterization of pathogenic and non-pathogenic JCPyV variants. It was of interest to determine if we could develop a triplex ddPCR in which JCPyV sequences could be detected simultaneously and together with the housekeeping gene RPP30. Using this reference gene is advantageous as it can serve as an indicator for both DNA quality and cellular quantity. Figure 4 is a representative two-dimensional descriptive ddPCR plot from the urine of a PML patient demonstrating JCPyV-DNA positivity for the JCPyV archetype virus, the non-virulent variant. Urine samples were again used for assay optimization because presence of the JCPyV archetype variant is defined by the demonstration of both large T-Ag and NCCR sequence target. The x-axis shows the amplitude on the VIC channel amplifying both the NCCR of the JCPyV archetype variant and the housekeeping gene RPP30 (lower right quadrant, green droplets) with a clear separation between the two targets. The y-axis shows the amplitude on the FAM channel amplifying JCPyV large T antigen (upper left quadrant, blue droplets). The black droplets in the lower left quadrants are negative droplets for all targets. Even though the pathogenic, prototype variants are predominant in the CSF of PML patients, the archetype virus could also be detected in some CSF specimens. Figure 5A shows representative plots of the triplex ddPCR applied to a CSF sample from a PML patient who has both JCPyV variants. This triplex positive sample was further confirmed by a singleplex ddPCR assay, which targeted only one genomic sequence per axis (Figure 5B, T-Ag; Figure 5C, NCCR and Figure 5D, RPP30).

Triplex ddPCR was performed on serum samples collected from the different cohorts including 20 healthy controls, 20 treatment-naïve patients with MS, 14 patients with MS undergoing treatment with natalizumab (Table 3) and 6 PML patients (Table 4). None of the sera from 20 HC or 34 MS patients had detectable viral DNA. Of the six PML patients, five had detectable JCPyV DNA in sera (Table 4).

### 3.4. Detection of JCPyV Viremia

The high sensitivity of ddPCR for detection of JCPyV in CSF prompted us to investigate whether the assay might have value as a screening tool for assessment of risk for PML.

The JCPyV antibody index is routinely used in the clinical setting as a biomarker to differentiate PML risk in patients who are under natalizumab treatment with no previous use of immunosuppressants. We employed our JCPyV triplex ddPCR methodology for the detection of JCPyV DNA in serum from three MS patients treated with natalizumab that had seroconverted (Supplementary Table S4). The presence of antibodies to JCPyV was determined by Focus Diagnostic where an index value was calculated based on a JCPyV antibody ELISA kit [22]. One patient had an index value of 0.44, while the other two had an index value equal to 2.37 and 1.27 respectively. The two patients with the higher JCPyV antibody index (MS1 and MS3) discontinued NTZ infusions after 48 months, while patient MS2 remained on NTZ treatment, but switched from the typical monthly infusion to extended interval dosing (EID), receiving NTZ at 6-week intervals (Q6), a strategy that has been suggested to reduce the risk of PML while still maintaining MS disease control [23]. Longitudinal serum samples were collected from these patients to determine if JCPyV DNA sequences could be amplified. JCPyV DNA remained undetected from all MS patients’ sera despite JCPyV seroconversion during the period of study, while the RPP30 housekeeping gene was amplified, indicating optimal DNA quality and cellular integrity (Supplementary Figure S3). None of these patients have developed PML. This is in contrast to patients with diagnosis of PML in whom JCPyV DNA was detected in serum by the ddPCR triplex assay from 5 of the 6 patients (Table 4).

Using the triplex ddPCR in the PML patients, it was possible to distinguish between the two JCPyV variants. Interestingly, the sera of two PML patients (PML 2 and PML 5) showed the presence of both the prototype, pathogenic variant, and the archetype, non-pathogenic variant. This was determined by the amplification of the large T-Ag copy number that was more than double the copy number of the NCCR specific to the archetype variant. Two other sera samples (PML 3 and PML 6) had detectable copies of viral DNA measured by amplification of only JCPyV-DNA of large T-Ag, indicating the presence of the rearranged, pathogenic variants. However, the serum of patient PML 4 was positive for both JCPyV-DNA large-T-Ag and NCCR with comparable copy numbers, indicating the presence of the non-pathogenic archetype variant only. Of interest was the observation in PML 1, whose underlying condition for PML was MS. PML patient 1 was on NTZ treatment for his MS for a total of 72 months, and this patient was also the only PML patient who had undetected JCPyV DNA in the serum (Table 4).

## 4. Discussion

In this study, we compared the sensitivity and specificity of a multiplex ddPCR assay with the gold standard, qPCR, to quantify the amount of JCPyV DNA in clinical samples. Together with MRI, the finding of JCPyV DNA in CSF is an established tool to confirm a diagnosis of PML [3]. Our data demonstrated that the ddPCR methodology is comparable in terms of precision to the qPCR. Because quantification of viral load with qPCR is dependent on extrapolation from a standard curve, this assay often results in high inter-assay variability and, at times, difficulty in accurately measuring the viral load, particularly in compartments with low numbers of cells, such as the CSF [12]. Therefore, qPCR is not optimal for viral load quantification, especially as consistency and reliability are essential for detecting biologically meaningful thresholds and changes. We employed a novel and now widely used technique, digital droplet PCR (ddPCR), that allows for the direct absolute quantification of a target gene in a given sample. Due to the random, independent segregation of DNA fragments into droplets, ddPCR uses Poisson algorithms to determine absolute copy numbers independently of a standard curve [15]. We have used ddPCR for the detection and quantification of viruses in biological samples including, HTLV-1 [24], HHV-6 [20], EBV [25,26] and CMV [25]. In this study, we examined the reliability of ddPCR in quantifying JCPyV in the CSF cells of patients with PML.

A comparison of 62 CSF total samples showed no statistical differences between qPCR and ddPCR for the detection of JCPyV. While analytical sensitivity is not the main focus of this report, ddPCR typically gave higher JC viral loads than qPCR but in group analysis this did not reach statistical significance (*p* = 0.057; Figure 3A). In addition, using standardized plasma samples analyzed for JCPyV by qPCR in 42 laboratories in the United States and Europe, no differences were observed between qPCR and ddPCR. Rather, we observed that ddPCR, due in part to the use of Poisson statistics, was more sensitive than qPCR, particularly at low copy numbers. This underscores a major benefit of ddPCR over qPCR: its capacity to resolve rare events and access targets at low template concentrations. This was evident in this study in which we demonstrated that qPCR could not report the viral loads in 15% of all PML clinical samples (9/62), since these samples were below the limit of detection for the qPCR assay (<250 copies/mL). By contrast, ddPCR was able to quantify all of the PML samples. Even more importantly, in 8% of these CSF samples (5/62), JCPyV was below the limits of detection and reported negative by qPCR, but were shown to be positive by ddPCR, consistent with clinical and radiological observations. This has significant clinical implications for the use of PCR as a diagnostic tool for the detection of JCPyV in CSF samples of patients with neurologic disease in which PML is being considered. The failure to detect JCPyV can postpone the diagnosis and, consequently, treatment options. The use of ddPCR to detect JCPyV DNA has also been recently described [27] and the authors further underline the high sensitivity of this technology to measure JC viral DNA. The use of highly sensitive and reliable PCR assays like ddPCR have also been reported in other clinical settings, such as SARS-CoV-2 infection [28,29,30]. These papers demonstrate the capability of ddPCR to detect SARS-CoV-2 in clinical samples earlier than qPCR, thereby reducing the number of false negative results. ddPCR could be a powerful complement to the standard qPCR and thus provide tremendous value during the COVID-19 pandemic [29]. A limitation of our study is the lack of extensive evaluation of the diagnostic specificity. However, we analyzed the JC viral load in 12 CSF from non-PML control samples, all of which had undetectable JCPyV. These data are a preliminary evaluation to address the point of assay specificity.

Another feature of the ddPCR methodology is the ability to detect more than two different targets in the same test. Previously, we have used this amplitude-based approach to characterize the coinfection of human herpesviruses 6A (HHV-6A) and HHV-6B by ddPCR [17,20]. In this present report, we developed a triplex ddPCR to simultaneously detect the presence of two JCPyV variants; the rearranged, prototype virulent variant and the archetype, non-virulent variant together with a control RPP30 housekeeping gene. We used different concentrations of primers and probes to detect distinct JCPyV targets that could be identified by unique fluorescence amplitudes. Viral DNA from cell-free samples such as serum, plasma and CSF were quantified in copies per mL in which RPP30 was not considered in the calculation. However, the use of the housekeeping gene as an internal control for the number of cells and DNA quality extends this ddPCR multiplex application to additional clinical samples, such as brain tissues or other organs. Specifically, the detection of RPP30 together with the different JCPyV variants allows for the quantification of the viral DNA per concentration of cells.

The presence of JCPyV DNA was analyzed in serum samples from HC, MS patients with and without natalizumab treatment and PML patients. Reports on the prevalence of JCPyV positive serum or plasma among these patients are inconsistent; the frequency of viral DNA in those compartments, particularly in MS patients treated with natalizumab, has been reported to vary from 0.3% (4/1397) and 1% (2/201) [31] to 15% and almost 40% in other reports [32,33]. In this study, JCPyV DNA was detected in five of the six PML sera using our triplex ddPCR, but undetected in the HC or MS patient cohorts, consistent with some previous reports [33,34]. Although in this small cohort, natalizumab treatment in MS patients was not associated with an increased prevalence of JCPyV viremia as reported by others [31], supporting data showing that routine clinical testing for JCPyV DNA in blood or urine it is not a beneficial method to predict PML risk in MS patients treated with natalizumab [31].

For the five PML patients in our study with JCPyV DNA detected by ddPCR in sera, we were able to quantify the presence of both JCPyV variants. Interestingly, one out of five (20%) demonstrated the presence of the archetype, non-pathogenic JCPyV variant, while two of five (40%) showed only the prototype, virulent JCPyV variant. The other two PML patients had both variants in serum as detected by ddPCR. Variable detection of one or both JCPyV variants in serum is consistent with our current understanding of JCPyV pathophysiology, with lymphoid organs being the likely predominant site of genomic conversion (2).

Timely diagnosis of PML is essential. The care of these individuals relies on the development and use of technologies that can accurately quantify JCPyV DNA in clinical samples to aid in early diagnosis, when CSF copy numbers are often below the limit of detection of qPCR. ddPCR for JCPyV is a powerful molecular tool that provides highly sensitive, reproducible and accurate quantification and JCPyV variant characterization.

## Figures and Tables

**Table 1:** Demographic and clinical characteristics of the 31 PML patients. Approximate time between PML diagnosis and sampling is given in months, with a range indicating sampling over multiple time points.

Patient	Sex/Race	Age	PML Diagnosis (Year)	Approximate Time between PML Diagnosis and Sampling (Months)	Underline Disease
Patient 1	M/WC	55	2013	60	HIV
Patient 2	M/WC	56	2020	1	CVID
Patient 3	F/WC	63	2020	1–17	Ocular pemphigoid
Patient 4	M/WC	79	2020	6	CLL
Patient 5	F/WC	76	2020	1	Lymphoma
Patient 6	M/WC	61	2016	51–54	HIV
Patient 7	F/WC	74	2020	2–3	Lymphoma
Patient 8	M	53	2021	0	Unknown
Patient 9	M/WC	60	2021	Pre-symptomatic ^b^ -0	HIV
Patient 10	M/WC	32	2021	0–4	Primary Immunodeficiency, diffuse large B cell lymphoma
Patient 11	M/Unknown	22	2021	0–4	DOCK-8 deficiency
Patient 12	F/H	44	2018	0	Unknown
Patient 13	F/WC	48	2014	51	Immunodeficiency
Patient 14	M/BAA	53	2020 ^a^	7	HIV
Patient 15	M/WC	26	2021	1	X-linked hyper IgE syndrome
Patient 16	M/BAA	39	2021	1	HIV
Patient 17	F/WC	71	2019	6	Lymphoma
Patient 18	F/BAA	50	2021 ^a^	Pre-symptomatic ^b^	HIV
Patient 19	M/WC	74	2021	1	Lymphoma
Patient 20	F/BAA	51	2021	3–8	Sarcoidosis
Patient 21	M/WC	65	2021	2–3	Scleroderma
Patient 22	M/BAA	49	2020	10–12	HIV
Patient 23	M/WC	34	2021	1	Primary Immunodeficiency
Patient 24	F/WC	74	2021	0	CLL
Patient 25	M/H	44	2021	Pre-symptomatic ^b^ −1	HIV
Patient 26	F/WC	66	2021	1–2	Idiopathic
Patient 27	F/WC	64	2019	8	ICL
Patient 28	M/WC	52	2019	84	HIV
Patient 29	M/WC	33	2019	0	ALL
Patient 30	M/WC	56	2021	0	Lymphoma
Patient 31	M/WC	70	2021	0	Lymphoma

^a^ “Possible PML” based on 2013 consensus diagnostic criteria; ^b^ “Pre-symptomatic” indicates the sampling was performed before the official PML diagnosis. M = Male; F = Female; WC = White/Caucasian; BAA = Black/ African American; H = Hispanic; HIV = Human Immunodeficiency Virus; CVID = Common Variable Immunodeficiency; CLL = Chronic Lymphocytic Leukemia; DOCK-8 = Dedicator of Cytokinesis 8; IgE = Immunoglobulin E; ICL = Interstrand Crosslinks ALL = Acute Lymphoblastic Leukemia.

**Table 2:** Comparison of ddPCR with qPCR proficiency data from cumulative results from a total of 42 clinical laboratories in the United States and Europe. SD = Standard Deviation.

Sample Code	qPCR Cumulative Results Log10[Copies Number] (SD)	ddPCR Log10[Copies Number]
01	2.9 (0.74)	3
02	3.5 (0.69)	3.9
03	3.1 (0.41)	3.5
04	4.4 (0.69)	4.6
05	4.2 (0.39)	4.8

**Table 3:** Healthy controls and MS patient cohorts’ characteristics.

Characteristic	Normal Control n = 20	MS Patients Non-Natalizumab Treated n = 20	MS Patients Natalizumab Treated n = 14
Sex M/F	6/14	6/14	2/12
Mean age ± SD (year)	49 ± 14.5/44 ± 15	54 ± 7/48 ± 13.1	43 ± 3.5/34 ± 7.4
Mean disease duration ± SD (year)	NA		
Type of MS (n)			
Relapsing-remitting (RRMS)	NA	17	14
Secondary progressive (SPMS)	NA	0	0
Primary progressive (PPMS)	NA	1	0
RIS	NA	2	0

**Table 4:** PML patients and JCPyV viremia. PML 1 is serum from a patient with MS/PML treated with natalizumab for 72 months. PML 2-6 are sera from PML patients with different underlying diseases.

ID	Sex	Race	Diagnosis	ddPCR T-Ag c/mL	ddPCR NCCR c/mL	JCPyV Titer	Natalizumab Start Date	Natalizumab End Date	Natalizumab Duration
PML 1	M	W/C	RRMS/PML	Und	Und	High	2011	Jun 2017	72 months
PML 2	M	H	CLL/PML	449	70	N/A	-	-	-
PML 3	M	W/C	CLL/PML	410	Und	N/A	-	-	-
PML 4	M	W/C	CLL/PML	277	171	N/A	-	-	-
PML 5	F	W/C	PID/PML	7304	1195	N/A	-	-	-
PML 6	F	W/C	Lymphoma/PML	158	Und	N/A	-	-	-

M = Male; F = Female; WC = White/Caucasian; H = Hispanic; RRMS = Relapsing Remitting Multiple Sclerosis; PML = Progressive Multifocal Leukoencephalopathy; Und = Undetected; CLL = Chronic Lymphocytic Leukemia; PID = Primary Immunodeficiency Disorder.

## Supplementary Materials

The following supporting information can be downloaded at: https://www.mdpi.com/article/10.3390/v14061246/s1, Table S1: Demographics of the 12 non-PML patients; Figure S1: Schematic of JCPyV genome; Table S2: Comparison of qPCR and ddPCR detectability; Table S3: Comparison of large T-Ag coding sequence copy numbers identified by qPCR and ddPCR; Figure S2: Agreement plot of log10 qPCR vs log10 ddPCR samples; Table S4: Characteristic of three patients in the MS-natalizumab cohort and JCPyV seropositive; Figure S3: Representative plots of triplex ddPCR of a serum sample from an MS patient.
