# Supplementary material for: Comparison of qPCR with ddPCR for the Quantification of JC Polyomavirus in CSF from Patients with Progressive Multifocal Leukoencephalopathy
Source: Viruses. 2022 Jun 8;14(6):1246. doi: 10.3390/v14061246 (PMC9229850; doi:10.3390/v14061246)
Supplement: Supplementary file 1 [file viruses-14-01246-s001.zip › viruses-1705482-supplementary.pdf]

Supplementary Materials

Supplementary Table S1. Demographic and clinical characteristics of 12 non-PML patients.

Patient	Sex/Race	Age	Diagnosis
Patient 1	M/Unknown	21	Optic neuritis, vertigo
Patient 2	M/Multiple Race	27	CSF Lymphoma, EBV positive
Patient 3	F/WC	49	Cryptococcal meningitis
Patient 4	M/WC	53	Sarcoma
Patient 5	F/WC	47	No known neurologic disease
Patient 6	M/WC	7	Neurologic deficiency, non-infectious
Patient 7	F/WC	53	Toxoplasmosis
Patient 8	M/WC	69	CNS Lymphoma EBV positive
Patient 9	F/WC	22	Neurologic deficiency, non-infectious
Patient 10	M/Hawaiian/Pacific Island	17	Glioblastoma
Patient 11	F/Unknown	52	RRMS
Patient 12	F/WC	72	MS Spectrum

M = Male; F = Female; WC = White/Caucasian; CSF = Cerebrospinal Fluid; EBV = Epstein-Barr Virus; CNS = Central Nervous System; RRMS = Relapsing-Remitting Multiple Sclerosis; MS = Multiple Sclerosis

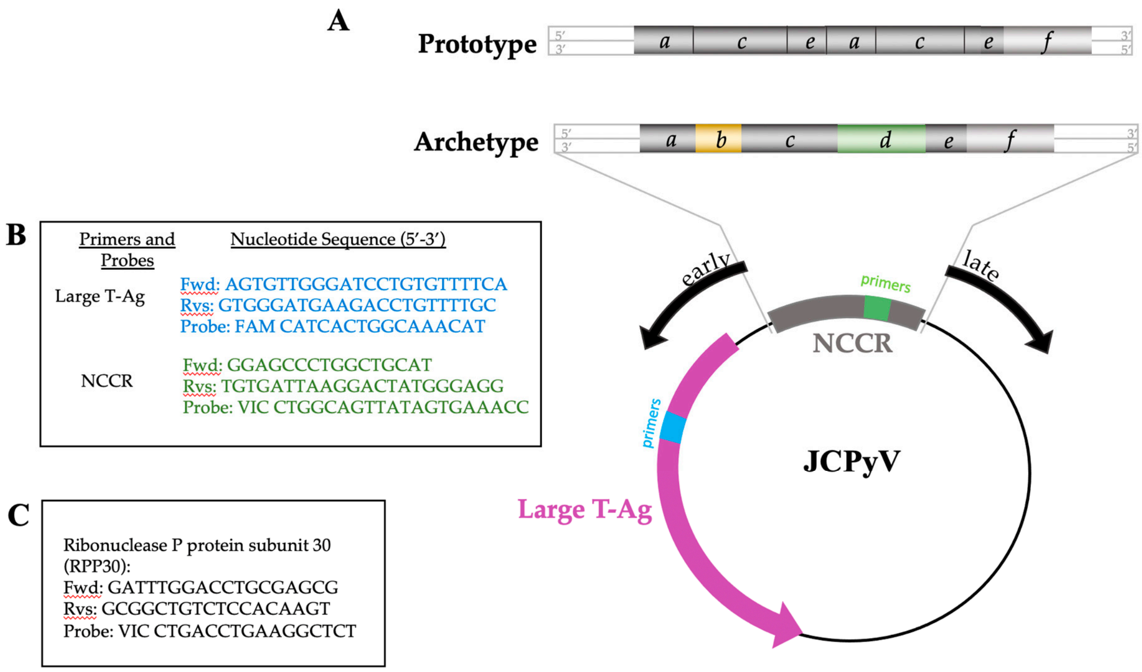

Supplementary Figure S1. Schematic comparison between JCPyV prototype and archetype genomes. The blue and green squares in the JCPyV schema identify the location of the primers/probe sets respectively in the Large T-Ag region and in the NCCR. The full sequence of the primers/probe sets is reported in the inset (B). The sequence of the primers and probe for the reference gene, RPP30, is reported in the inset (C).

Supplementary Table S2. Direct comparison of numbers of qPCR detectability versus ddPCR detectability. Out of 54 ddPCR samples with detectable JCPyV levels, 5 were undetectable on qPCR.

	**Detectable ddPCR**	**Undetectable ddPCR**	
**Detectable qPCR**	49	0	49
**Undetectable qPCR**	5	8	13
	54	8	62

Supplementary Table S3. Comparison of large T-Ag coding sequence copy numbers identified by qPCR and ddPCR

Patient (date)	ddPCR copies/mL	qPCR copies/mL
1 (08/06/19)	766	1600
2 (03/05/20)	8.50E+06	6.74E+06
2 (03/25/20)	2.10E+06	9.32E+05
3 (06/26/20)	1.59E+04	2350
3 (07/14/20)	1.19E+04	1100
3 (08/18/20)	1031	350
3 (09/01/20)	281	650
3 (11/03/20)	594	600
3 (02/16/21)	422	<250
3 (05/18/21)	Und	Und
3 (10/13/21)	Und	Und
4 (09/03/20)	391	<250
5 (09/09/20)	7188	4300
6 (01/21/21)	500	<250
6 (04/20/21)	1109	300
7 (03/01/21)	1.09E+04	3600
7 (03/18/21)	1172	350
7 (04/12/21)	3594	1500
7 (04/23/21)	4218	600
8 (03/19/21)	4.30E+05	2.28E+05
8 (04/12/21)	4.70E+04	3.06E+04
9 (01/27/21)	344	Und
9 (03/19/21)	391	<250
9 (03/30/21)	1171	<250
10 (04/30/21)	1219	550
10 (06/01/21)	2156	1350
10 (06/28/21)	438	600
10 (08/05/21)	625	1750
11 (06/24/21)	1234	350
11 (07/14/21)	3031	Und
11 (08/05/21)	5313	2800
11 (09/02/21)	4031	4450
11 (10/26/21)	1625	600
12 (11/08/18)	356	Und
13 (06/08/18)	178	Und
14 (02/05/21)	Und	Und
15 (06/09/21)	4063	700
16 (07/13/21)	2.00E+06	8.95E+05
17 (07/30/19)	6.59E+04	1.73E+04
18 (01/12/21)	Und	Und
19 (08/26/21)	828	<250
20 (07/08/21)	7.73E+04	1.55E+04

20 (08/04/21)	1.36E+05	3.31E+04
20 (09/23/21)	1359	2560
20 (12/10/21)	2188	1700
21 (04/12/21)	281	<250
21 (05/17/21)	Und	Und
22 (08/26/21)	766	Und
22 (11/16/21)	Und	Und
22 (12/22/21)	Und	Und
23 (10/05/21)	2.28E+04	6650
24 (08/25/21)	2.41E+04	1.38E+04
25 (10/5/21)	Und	Und
25 (11/12/21)	266	<250
25 (12/03/21)	1281	250
26 (09/30/21)	2531	1100
26 (10/19/21)	1219	1050
27 (09/05/19)	1687	<250
28 (11/06/19)	1859	300
29 (11/15/19)	1125	450
30 (10/21/21)	31250	23700
31 (12/17/21)	656	600

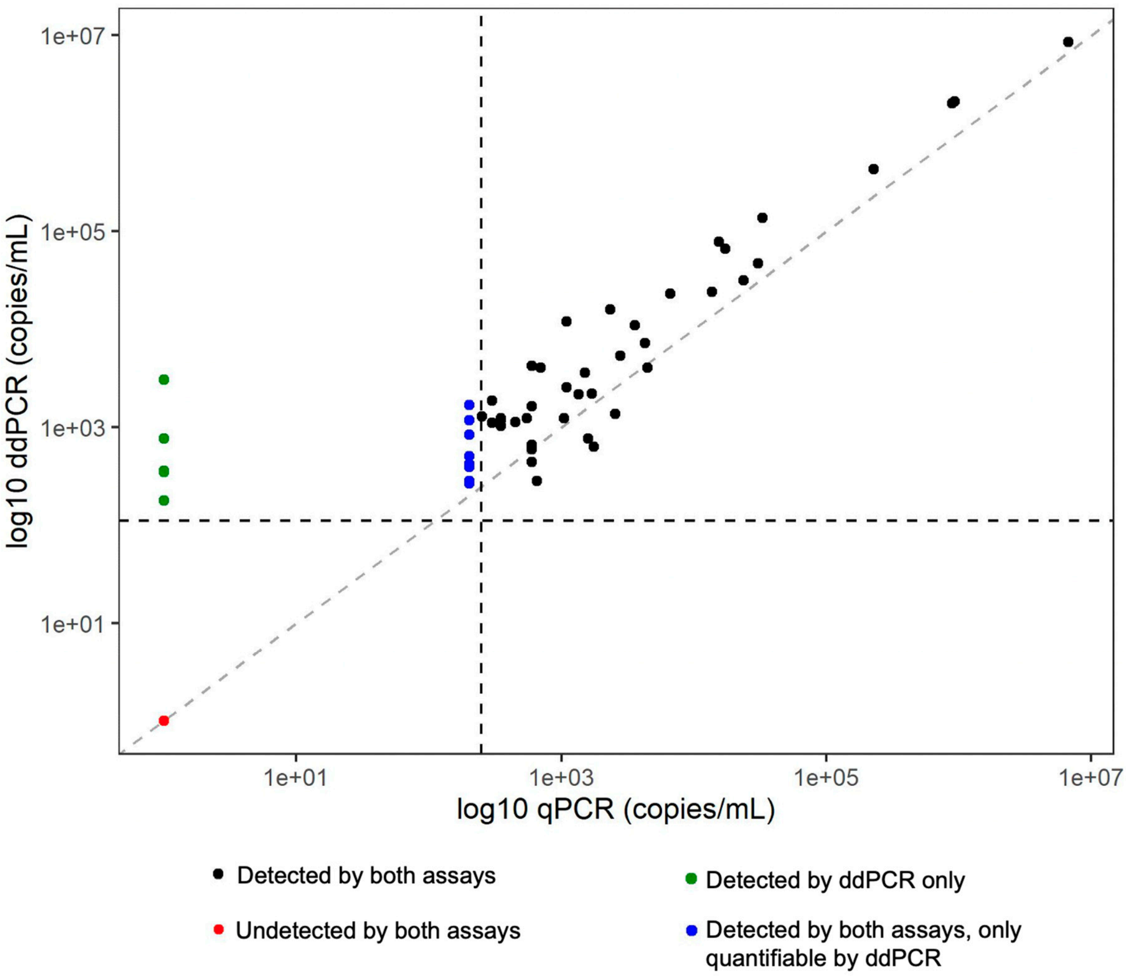

Supplementary Figure S2. Agreement plot of log10 qPCR vs log10 ddPCR samples. Observations above the gray dashed identity line indicate samples where higher levels of ddPCR were detected compared to qPCR. The dashed black vertical line indicates the cutoff for detectability and ability to be quantified in qPCR (>250). The dashed black horizontal line indicates the cutoff for detectability in ddPCR (110). There are a total of 8 samples that were undetectable on both methods, shown as one overplotted point in red.

Supplementary Table S4. MS-natalizumab treated patients, JCPyV seropositive

ID	Natalizumab Duration	JCPyV Ab-Titer
MS 1	48 months	2.37
MS 2	Still on; Q6	0.44
MS 3	48 months	1.27

MS = Multiple Sclerosis; Q6 = 6-week interval

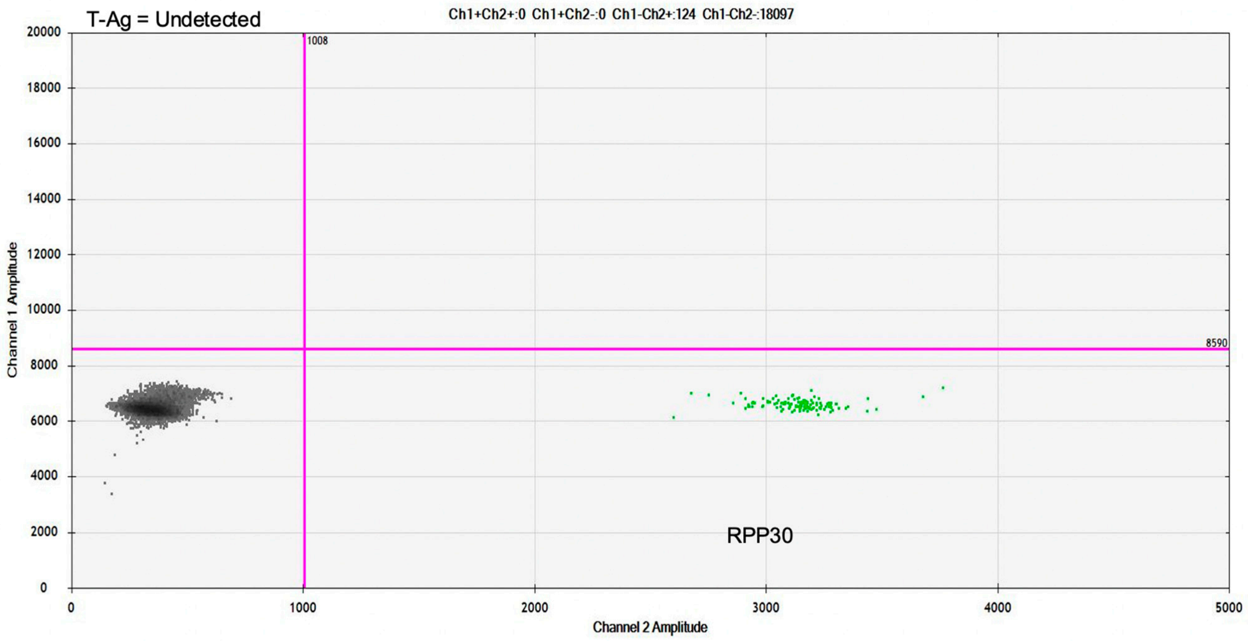

Supplementary Figure S3. Representative plots of triplex ddPCR of a serum sample from an MS patient. JCPyV genomes were undetected while only the RPP30 housekeeping gene was amplified
